# Generative Artificial Intelligence Transitions Pharmaceutical Development from Empirical Screening to Predictive Molecular Design and Clinical Trial Optimization

**DOI:** 10.3390/ph19040614

**Published:** 2026-04-13

**Authors:** Ghaith K. Mansour, Hatouf H. Sukkarieh

**Affiliations:** 1 College of Pharmacy, Alfaisal University, Riyadh 11533, Saudi Arabia; gkmansour@alfaisal.edu; 2 Department of Pharmacology, College of Medicine, Alfaisal University, Riyadh 11533, Saudi Arabia

**Keywords:** generative artificial intelligence, drug discovery, de novo molecular design, clinical trial optimization, precision medicine, deep learning, pharmacogenomics, synthetic control arms

## Abstract

The traditional paradigm of pharmaceutical research is characterized by substantial inefficiency, requiring extensive timelines and billions of dollars while suffering from high clinical attrition rates. The integration of generative artificial intelligence (AI) is driving a paradigm shift from empirical experimentation toward predictive, data-driven innovation. This review evaluates state-of-the-art applications of these technologies across the drug discovery and development pipeline. By analyzing multi-omics data streams, AI models can elucidate complex disease mechanisms and identify novel therapeutic targets. Deep generative architectures facilitate the algorithmic creation of novel molecular entities, enabling the design of therapeutics with complex polypharmacological profiles. Furthermore, AI is enhancing the clinical testing phase through large language models (LLMs) that improve patient enrollment and through synthetic control arms (SCAs) that provide computational alternatives to traditional placebo groups. Despite these advances, the scientific community must address inherent algorithmic biases stemming from demographic underrepresentation and mitigate the risks of data hallucinations. Ultimately, realizing the full translational potential of generative AI in precision medicine may require the widespread adoption of explainable AI (XAI) frameworks and rigorous data standards.

## 1. Introduction

The traditional paradigm of pharmaceutical research and development is characterized by high degrees of inefficiency, substantial capital expenditures, and significant clinical attrition rates. Historically, the journey of a novel therapeutic from initial target identification to regulatory approval spans 10 to 15 years, with overall research and development expenses frequently exceeding $2 billion per approved drug [1]. Furthermore, fewer than 10% of therapies entering Phase I clinical trials ultimately achieve market authorization [1,2]. This systemic inefficiency stems in part from the immense, multidimensional search space inherent to computational chemistry. The theoretical “chemical universe” is estimated to contain up to 10^60^ drug-like small molecules, presenting a combinatorial challenge that traditional high-throughput screening and heuristic-based medicinal chemistry cannot efficiently navigate [3,4].

The integration of AI, and more specifically deep generative modeling, is driving a paradigm shift in pharmaceutical sciences. Rather than relying solely on empirical trial-and-error experimentation, AI pipelines are increasingly influencing multiple stages of the pharmaceutical value chain [5,6]. Deep learning, machine learning, and natural language processing (NLP) models are now capable of processing massive multi-omics datasets, generating novel molecular entities de novo, predicting complex protein-ligand binding affinities, and optimizing clinical trial designs with improved precision [1,7]. This transformation may not only accelerate the speed of discovery but also alter the nature of the molecules being discovered, potentially enabling the design of therapeutics tailored for complex polypharmacological profiles and specific patient subpopulations [6,8].

To integrate these advances within a unified analytical structure, this review employs the Generative AI Continuum as an organizing framework, a systems-level conception of the pharmaceutical pipeline in which multi-omics target identification, generative molecular design, ADMET and binding affinity prediction, biomarker discovery, and clinical trial optimization are treated not as isolated methodological domains but as sequentially interdependent stages of a single data-driven value chain. This framework is developed across Section 2, Section 3, Section 4, Section 5 and Section 6 and synthesized in Section Toward a Self-Improving Pharmaceutical Pipeline, where its translational implications and research priorities are articulated.

This review evaluates state-of-the-art applications of generative AI across the drug discovery and development pipeline. It begins by examining the role of AI in elucidating disease mechanisms and identifying novel therapeutic targets through multi-omics integration. The narrative then transitions into a mechanistic analysis of generative architectures—including diffusion models, variational autoencoders (VAEs), and generative adversarial networks (GANs)—currently utilized for de novo molecular design, three-dimensional (3D) conformation generation, and lead optimization. The analysis subsequently explores how AI is influencing biomarker discovery and clinical trial optimization through the deployment of LLMs and SCAs. Furthermore, this review assesses the inherent limitations, algorithmic biases, and infrastructural challenges that must be addressed to realize the translational potential of AI in precision medicine. Finally, this review provides an original analytical contribution beyond descriptive summarization, this review proposes a unifying perspective: the “Generative AI Continuum.” Rather than treating target identification, molecular design, and clinical trial optimization as isolated computational subfields, we argue that generative AI is enabling their convergence into a single, iteratively self-improving epistemic cycle. In this cycle, multi-omics knowledge graphs condition molecular generation; generated molecular candidates inform synthetic biomarker hypotheses; and synthetic control arm architectures feed real-world pharmacological signal back into target validation models. This bidirectional information flow from biology to chemistry to clinical outcome and back represents a qualitative departure from the unidirectional empirical pipeline and constitutes the central thesis of this review. Where prior reviews have catalogued AI applications within pipeline stages, we seek to critically examine the evidence for and against this integrative vision, identifying where the Continuum is already operational, where it remains aspirational, and what methodological, regulatory, and equity barriers must be resolved before it can be responsibly deployed at scale.

## 2. AI-Driven Target Identification and Disease Mechanism Elucidation

The foundational step of modern therapeutic discovery is the precise identification and rigorous validation of molecular targets whose modulation yields a predictable and safe therapeutic effect. Historically, target discovery relied heavily on experimental comparative profiling methods, such as stable isotope labeling by amino acids in cell culture (SILAC; Thermo Fisher Scientific, Waltham, MA, USA) and genome-wide association studies (GWAS) [9]. While these empirical methods have uncovered critical biological pathways for monogenic and structurally straightforward diseases, they are resource-intensive and often struggle to capture the non-linear, multidimensional interactions inherent in complex, multifactorial diseases such as idiopathic pulmonary fibrosis (IPF) and Alzheimer’s disease (AD) [9,10]. AI methodologies have expanded the analytical toolkit by integrating heterogeneous multi-omics data streams—encompassing genomics, transcriptomics, proteomics, epigenomics, and metabolomics—to construct predictive systems-biology models of disease pathology [10,11].

### 2.1. Multi-Omics Integration and Deep Knowledge Graphs

Modern target identification leverages massive knowledge graphs and deep learning algorithms to synthesize structured biological data with unstructured data, such as scientific literature and global patent repositories [10]. By embedding genes, proteins, diseases, and chemical compounds into a shared high-dimensional latent space, AI models can infer previously unrecognized relationships between specific molecular targets and phenotypic disease expressions. These models utilize advanced graph neural networks (GNNs) and NLP to identify “druggable” targets that exhibit high topological significance within disease-associated protein–protein interaction (PPI) networks [12,13].

This analytical capacity is particularly valuable in under-characterized and orphan disease states. For instance, in the domain of IPF, a devastating fibrotic disease with high mortality rates.AI-driven target discovery platforms have processed multi-omics datasets derived from patient tissues alongside millions of text documents from the scientific literature [1,14]. Through deep feature extraction and mechanism-of-action filtering, algorithms prioritized TRAF2- and NCK-interacting protein kinase (TNIK) as a promising, first-in-class target for pulmonary fibrosis [14,15]. While TNIK had been previously implicated in certain oncology indications via the Wnt/β-catenin signaling pathway, its specific role in pulmonary fibrogenesis was identified through the platform’s multi-omics inference and AI-driven analysis [1,15].

### 2.2. Clinical Validation of AI-Identified Targets

The capability of generative models to propose novel targets is being substantiated by clinical validation. The discovery of the TNIK target directly catalyzed the development of Rentosertib, a small molecule inhibitor designed de novo via generative chemistry engines [15]. The end-to-end timeline from project initiation and target discovery to preclinical candidate nomination reportedly required approximately eighteen months, at an estimated out-of-pocket cost of approximately $150,000—although these figures are industry self-reported and should be interpreted with caution [1,14]. In 2024, Rentosertib became one of the first AI-designed drugs, modulating an AI-discovered target, to achieve proof-of-concept success in a Phase IIa randomized, double-blind, placebo-controlled clinical trial [15,16]. Patients receiving the optimal dose demonstrated a statistically significant improvement of +98.4 mL in improvements in forced vital capacity (FVC) versus placebo, providing initial clinical evidence supporting the computational hypothesis [15,16].

### 2.3. Expanding Target Discovery to Neurodegeneration

Beyond fibrosis, AI-driven target identification is being actively deployed against neurodegenerative conditions, notably AD and AD-related dementias (ADRD). The historically high clinical attrition rate in AD trials has been partially attributed to an incomplete understanding of the disease’s underlying pathophysiology [12]. In response, research groups are leveraging machine learning to map the complex interactome of the aging brain [12,13]. Contemporary frameworks apply Bayesian algorithms and multi-omics integration to infer AD risk genes directly from GWAS loci, dynamically mapping these loci to human PPI networks to identify therapeutic targets [13]. By synthesizing evolving models of tauopathy, amyloidosis, and neuroinflammation, AI enables the stratification of disease progression into mechanistic pathways, potentially accelerating early drug discovery efforts in neurodegeneration [12].

## 3. Generative Artificial Intelligence in De Novo Molecular Design

Once a viable biological target is identified, the challenge transitions to discovering or designing a chemical entity that can bind to the target with high affinity, stringent selectivity, and an optimal safety profile. Traditional high-throughput screening involves physically or virtually screening millions of existing compounds from predefined chemical libraries [5]. However, this method is fundamentally limited by the specific chemical space contained within those pre-existing libraries, leaving the vast majority of the 10^60^ possible drug-like molecules unexplored [3,4]. Generative AI addresses this limitation by shifting the paradigm from screening existing compounds to the algorithmic creation of novel matter [5]. Rather than searching for a rare entity in a predefined dataset, generative models learn the fundamental topological and quantum grammar of chemistry, enabling them to generate optimized molecules entirely de novo [4].

### 3.1. Evolution of Deep Generative Architectures

The field of de novo molecular generation utilizes several distinct classes of deep learning architectures, each offering unique mathematical approaches to sampling the continuous chemical latent space. Table 1 summarizes the foundational mechanisms and advantages of these architectures.

A critical comparative evaluation of these architectures reveals important performance tradeoffs that are often obscured in descriptive reviews. Geometric diffusion models—exemplified by DiffSBDD and TargetDiff—currently achieve state-of-the-art performance on structure-based drug design benchmarks such as the CrossDocked2020 dataset, reporting Vina docking scores averaging −7.5 kcal/mol and drug-likeness (QED) scores of approximately 0.48, outperforming autoregressive graph models and VAE-based approaches on both metrics [3,4,17,18]. However, diffusion models carry a substantial computational cost: generating a single optimized 3D ligand within a protein binding pocket can require 500–1000 reverse diffusion steps, making high-throughput virtual screening computationally prohibitive without hardware acceleration [17,19]. VAEs, by contrast, provide efficient continuous latent space interpolation amenable to multi-parameter property optimization, but suffer from posterior collapse in high-dimensional molecular spaces, resulting in chemically valid but structurally redundant outputs [20,21]. GANs offer fast sampling but are notoriously difficult to train stably in molecular generation tasks, exhibiting mode collapse in which the generator converges on a narrow subset of the accessible chemical space [22,23]. Normalizing flows provide exact likelihood estimation—a significant theoretical advantage for hit rate optimization—but scale poorly to large, flexible molecules due to the computational complexity of maintaining invertible transformations across hundreds of atoms [24,25]. These tradeoffs suggest that no single architecture is universally optimal; rather, rational pipeline design requires matching architecture class to the specific optimization objective, target class, and computational resource envelope available [4,19,23].

### 3.2. Molecular Representations: From Linear Strings to Geometric Graphs

The efficacy of any generative model is tightly coupled to how molecules are computationally represented as input and output. Early generative models relied predominantly on linear one-dimensional (1D) textual strings, most notably the Simplified Molecular Input Line Entry System (SMILES, Daylight Chemical Information Systems, Aliso Viejo, CA, USA) [3,8]. While efficient for computational processing, SMILES representations often suffer from structural fragility; a minor alteration of a single character in a sequence can result in topological invalidity, such as open structural rings or impossible atomic valences [3]. To address this vulnerability, the cheminformatics field developed Self-Referencing Embedded Strings (SELFIES, Open-source; developed by Alán Aspuru-Guzik lab, University of Toronto, Toronto, ON, Canada), which rely on a semantically constrained mathematical grammar guaranteeing that every generated string corresponds to a chemically valid molecule [3]. Subsequently, fragment-based representations such as SAFE, GroupSELFIES, and fragSMILES were introduced to better capture chemically rich features and stereochemical chirality [3].

Despite these improvements in linear text representations, molecular binding is fundamentally a 3D spatial and thermodynamic phenomenon. Consequently, state-of-the-art generative design has transitioned toward two-dimensional (2D) graph representations—where atoms function as nodes and chemical bonds as edges—and, increasingly, toward 3D point clouds, which encode precise spatial coordinates and atomic torsional angles [3,4]. Processing these complex geometric representations requires advanced architectures such as Equivariant Graph Neural Networks (EGNNs) [3,4]. These neural networks are mathematically designed to be roto-translationally equivariant, meaning the network’s understanding of the molecule remains consistent regardless of how the 3D structure is rotated or translated in space [3,4].

### 3.3. Geometric Diffusion Models for 3D Conformation Generation

A critical bottleneck in virtual screening and structure-based drug design is predicting the diverse, energetically favorable 3D conformations a flexible 2D molecular graph can adopt. The biological activity and target affinity of a drug are dictated by its localized 3D conformations. To address this, researchers have developed geometric diffusion models, including frameworks such as GeoDiff (Tsinghua University, Beijing, China) [2,26,27]. These generative systems operate by establishing a Markov chain that reverses a diffusion process; they initialize with random noise distributions of atomic coordinates and progressively denoise them to generate stable molecular conformations [26,28].

By framing conformation generation as a thermodynamic diffusion process, these models learn a localized, data-driven energy function from existing conformation databases [26,29]. Because the likelihood of conformations must remain roto-translationally invariant to be physically accurate, they utilize specialized equivariant Markov kernels to preserve the geometric integrity of the generated structures throughout the denoising trajectory [26,30]. Empirical results suggest that this geometric approach can outperform traditional molecular dynamics simulations and earlier machine learning methods in estimating the multimodal distribution of conformations, particularly for large, flexible drug-like molecules [26,31,32].

### 3.4. Polypharmacology and Multi-Target Therapeutic Design

Historically, the pharmaceutical industry pursued a “one-drug, one-target” philosophy. However, complex systemic diseases such as metastatic oncology and progressive neurodegeneration frequently exhibit network redundancy, pathway compensation, and adaptive resistance mechanisms, rendering single-target therapies less effective over time [8,33,34]. AI is enabling a shift toward rational polypharmacology—the deliberate design of single molecules that interact with multiple specific therapeutic targets simultaneously to address compensatory disease pathways [8,33,35].

Deep generative models, driven by reinforcement learning and active learning paradigms, are being deployed to construct multi-target agents. Frameworks utilizing unified, multi-objective reward schemes condition generative neural networks to explore the intersection of distinct pharmacological latent spaces, seeking molecules that satisfy conflicting binding constraints [8,36,37]. For example, AI models condition chemical structure templates with integrated biological insights, such as patient transcriptomic and genomic profiles, to generate molecules exhibiting dual activity against challenging cancer-relevant targets, including PI3K and BRD4 [8,38,39].

Emerging efforts are beginning to extend these generative architectures toward non-classical therapeutic modalities, including proteolysis-targeting chimeras (PROTACs), macrocycles, and covalent binders, though the scarcity of such compounds in foundational training corpora means that generative coverage of these modality spaces remains nascent relative to conventional small-molecule design [3,4].

## 4. Lead Optimization and Binding Affinity Prediction

The algorithmic generation of a novel molecular scaffold represents only the preliminary phase of therapeutic design. The subsequent phase, lead optimization, requires iterative modification of the molecule to maximize target binding affinity, ensure target selectivity, and optimize absorption, distribution, metabolism, excretion, and toxicity (ADMET) profiles [1,18,40]. In traditional pipelines, this is a notably slow, capital-intensive cycle of chemical synthesis and rigorous in vitro assaying [5,19].

### Physics-Informed Deep Learning in Affinity Prediction

To accelerate lead optimization, computational chemists have long relied on in silico simulation methods such as free energy perturbation (FEP) and molecular mechanics generalized Born surface area (MM-GBSA) calculations [19,41,42]. While accurate in calculating thermodynamic binding free energies, these simulation methods are computationally expensive, precluding their routine use in evaluating large libraries of generative outputs [19,41].

A notable example of the convergence between physics and AI is the Pairwise Binding Comparison Network (PBCNet), an architecture designed to predict the relative binding affinity among congeneric ligands [19]. This network utilizes a physics-informed graph attention mechanism that processes a pair of protein pocket-ligand complexes simultaneously, explicitly encoding the differential spatial and electrostatic interactions between closely related molecular analogs to achieve ranking ability that approaches FEP performance at substantially reduced computational cost [19,43].

A candid assessment of where AI genuinely outperforms physics-based methods and where it does not is essential for the field to set realistic expectations. Free energy perturbation (FEP) remains the gold standard for relative binding affinity prediction in congeneric series, achieving mean unsigned errors (MUEs) of approximately 0.8–1.0 kcal/mol in prospective benchmarking studies [19,41,42]. Machine learning-based binding affinity models such as PBCNet and Uni-Mol achieve MUEs of 1.0–1.3 kcal/mol on the same benchmark sets [19,44], representing a modest but acceptable accuracy loss in exchange for orders-of-magnitude improvement in throughput: FEP calculations for a single compound typically require 12–48 h on GPU clusters, while ML inference operates in milliseconds per compound [43,45]. Critically, however, the comparative advantage of AI narrows significantly when applied outside the training domain. Models trained on kinase-focused datasets demonstrate pronounced performance degradation when evaluated on GPCRs or allosteric sites, with MUE increases of 0.3–0.7 kcal/mol, revealing the fundamental limitation of data-driven approaches in low-data regimes [45,46]. Furthermore, physics-based methods retain an inherent mechanistic interpretability advantage: FEP produces thermodynamic cycle-decomposed energy terms that chemists can use to rationalize structural modifications, whereas neural network affinity predictions are often opaque to direct chemical reasoning [41,45]. These considerations suggest that hybrid architectures—in which ML models perform rapid triage of generative outputs and FEP is reserved for final lead validation—currently represent the most pragmatically rigorous approach to the lead optimization problem [19,43,45].

Similarly, deep learning models utilize advanced neural architectures—ranging from voxel grid-based 3D-Convolutional Neural Networks (3D-CNNs) to Graph Attention Networks employing radial atomic environment vectors—to predict protein-ligand binding affinities directly from structural coordinates [20,21,44]. To overcome labeled data scarcity in computational chemistry, self-supervised learning techniques are increasingly utilized, enabling predictive models to learn fundamental biophysical interactions from large unlabeled structural databases before fine-tuning on specific target affinities [21,44,47]. The integration of these predictive ADMET and affinity models into “closed-loop” automated synthesis laboratories represents an advancing frontier in autonomous drug discovery frameworks [8,22,48].

## 5. Biomarker Discovery and Multi-Modal Data Integration

The ultimate efficacy of a highly optimized therapeutic agent is dependent upon its administration to a biologically receptive and correctly diagnosed patient population. Thus, biomarker discovery—the identification of measurable indicators of pathogenic processes or pharmacological responses—is as critical as the drug design phase itself [1,49,50]. Generative AI and advanced machine learning models are enhancing the identification of clinical biomarkers by integrating ultra-high-dimensional, multimodal data streams including genomics, electronic health records (EHRs), wearable sensor data, and medical imaging [10,51].

### 5.1. Multi-Omics Frameworks for Precision Diagnostics

AI algorithms excel at detecting subtle, non-linear correlations across disparate datasets that human analysts may not perceive. Several computational frameworks illustrate this capability. The MILTON (AstraZeneca, Cambridge, UK) framework demonstrates that augmenting traditional clinical diagnostic data with AI-selected proteomics biomarkers can improve predictive performance across various disease states [10,52]. The PRSmix (Broad Institute of MIT and Harvard, Cambridge, MA, USA) framework utilizes machine learning (elastic net regression) to dynamically aggregate and optimize polygenic risk scores across complex populations, capturing epistatic interactions to improve genomic risk biomarkers [10,53]. The EpiSign (EpiSign Inc., London, ON, Canada) framework deploys support vector machines (SVMs) to analyze methylation data, identifying “episignatures” associated with Mendelian disorders [10,54,55]. The CytoTRACE2 (Stanford University, Stanford, CA, USA) framework utilizes an interpretable deep learning approach based on Gene Set Binary Networks to isolate cellular gene signatures capable of predicting individual responses to chemotherapy and immune checkpoint inhibitors in oncology [10,56]. Table 2 summarizes these frameworks [10,56].

### 5.2. Generative AI Agents as Virtual Laboratories

Beyond static predictive modeling, generative AI is evolving toward orchestrating the dynamic discovery process itself through advanced multi-agent systems. Recent studies have demonstrated the deployment of LLM-powered “Virtual Labs” in biological research [10]. Within these autonomous environments, independent AI agents assume specialized roles—such as computational biologist, immunologist, and principal investigator [10]. When tasked with developing novel nanobodies against emerging viral variants, these interacting agents collaboratively design computational pipelines, propose sequence modifications, execute in silico binding simulations, and finalize therapeutic candidate selections [10]. The output of this multi-agent process has been validated in wet-lab experiments, suggesting that AI can automate aspects of scientific hypothesis formulation and experimental protocol generation [10].

Furthermore, deep learning has contributed to the development of “deep aging clocks” within the longevity field [57]. Utilizing GANs and specialized LLMs, these clocks analyze chronological multi-omics data to estimate a patient’s biological age with high precision. These tools serve as biomarkers for tracking aging processes and may help identify “geroprotectors”—molecules capable of modulating biological aging pathways [57].

## 6. Clinical Trial Optimization and Synthetic Control Arms

Despite advances in preclinical discovery and molecular generation, the clinical trial phase remains one of the most rigorous, expensive, and failure-prone bottlenecks in pharmaceutical development [7]. Late-stage trials often fail not because the underlying drug mechanism is inactive, but due to suboptimal patient stratification, inadequate statistical powering, and flawed protocol design [2,58]. AI is addressing these challenges through advanced predictive modeling, LLM-based trial matching, and synthetic data architectures [7].

### 6.1. Large Language Models for Patient Screening and Enrollment

Patient recruitment is a pervasive challenge, often delaying critical trials by months or years. The stringent inclusion and exclusion criteria of modern precision oncology trials make identifying eligible participants arduous for human clinical coordinators. The advent of medical LLMs has introduced improved efficiency to this process. For instance, the TrialGPT (National Library of Medicine (NLM) and National Cancer Institute (NCI), National Institutes of Health (NIH), Bethesda, MD, USA) system—an application built upon advanced LLMs—was deployed to match complex patient health records against intricate trial criteria [59]. In large-scale evaluations spanning more than 75,000 clinical trials, TrialGPT achieved a patient-criteria matching accuracy of 87.3% and a recall rate exceeding 90% [59]. By rapidly analyzing unstructured EHRs and cross-referencing them with trial protocols, AI systems reportedly reduced patient screening time by 42.6%, performing at parity with or exceeding the accuracy of human clinical coordinators [59].

### 6.2. Data-Driven Stratification and Adaptive Trial Design

AI is simultaneously optimizing the design of trials themselves. Frameworks such as Trial Pathfinder (Stanford University, Stanford, CA, USA, in collaboration with Genentech, South San Francisco, CA, USA) represent a movement toward data-driven trial design [60]. By analyzing real-world data (RWD) and simulating trial outcomes under varying hypothetical parameters, AI can optimize inclusion and exclusion criteria [60]. Empirical studies utilizing this framework have demonstrated that algorithmically loosening specific, historically arbitrary criteria can increase the number of eligible patients without compromising the statistical integrity or safety of the trial. Optimizing patient selection through this approach has been reported to reduce the overall hazard ratio by approximately 0.05, suggesting that AI-stratified cohorts may yield stronger clinical efficacy signals [60].

Within personalized oncology, multimodal deep learning models can fuse temporal clinical data with molecular markers and radiological imaging to predict tumor progression within active trial participants [60]. These tools enable adaptive trial designs, wherein patient treatment allocation can be dynamically adjusted based on continuous biomarker feedback, thereby potentially minimizing the administration of ineffective treatments to non-responsive cohorts.

### 6.3. Synthetic Control Arms

One of the most potentially disruptive applications of generative AI in clinical development is the generation of SCAs [2,27,61]. In traditionally structured randomized controlled trials (RCTs), a portion of the cohort receives a placebo or standard-of-care treatment to provide a statistical baseline [2]. However, in severe, rapidly progressing indications such as late-stage oncology, IPF, or severe neurodegeneration, allocating patients to a placebo arm poses ethical dilemmas and hinders trial recruitment [2].

Generative AI provides a computational alternative to traditional placebo groups. Utilizing historical clinical trial data, RWD, and clinico-genomic databases, generative neural networks (frequently relying on architectures such as Conditional Restricted Boltzmann Machines) ingest baseline patient data to create “digital twins” of enrolled experimental patients [61,62]. By analyzing a patient’s baseline multi-omics and longitudinal clinical profile, the generative model simulates the trajectory of that patient’s disease progression under placebo conditions [2,62].

These in silico simulated trajectories form the SCA [2]. Because the underlying generative models can map high-dimensional causal relationships, they may account for hidden confounding variables that affect simple historical data comparisons [2]. By substituting empirical placebo patients with synthetic data proxies, trial sponsors can construct single-arm trials wherein recruited patients receive the experimental therapeutic, while the AI provides the statistical benchmark required for regulatory efficacy evaluations [2,27]. Furthermore, data generalization techniques and privacy-preserving algorithms help ensure that these synthetic datasets maintain statistical utility while minimizing the risk of sensitive patient information disclosure [63]. The adoption of SCAs has the potential to reduce trial costs, accelerate timelines, and provide an ethical alternative to traditional placebo designs, though regulatory acceptance remains an evolving area [2,64].

The regulatory acceptance of synthetic control arms (SCAs) depends critically on their demonstrated ability to replicate control arm outcomes with quantifiable fidelity. Current validation studies report that generative SCA models achieve concordance correlations (CCC) of 0.72–0.89 with historical placebo outcomes in oncology indications when trained on sufficiently large clinico-genomic databases, but performance degrades substantially in rare disease contexts where historical data are sparse, with CCC falling to 0.51–0.64 [2,27,61,62]. A critical source of error in SCA generation is temporal confounding: historical control populations are subject to secular trends in standard-of-care management that may not be captured in the generative model’s training distribution, leading to systematic underestimation or overestimation of placebo response rates [2,65]. In the IPF context specifically, the introduction of pirfenidone and nintedanib as standard-of-care agents in 2014 fundamentally altered placebo arm decline trajectories, rendering pre-2014 historical controls unreliable for contemporary SCA construction without explicit temporal recalibration [66,67]. Furthermore, the Conditional Restricted Boltzmann Machine architectures most commonly used for SCA generation assume conditional independence between feature sets that is biologically implausible in multi-morbid patient populations, potentially introducing unmeasured confounding that would not survive classical propensity score sensitivity analysis [62,63]. These methodological limitations underscore why regulatory agencies currently require SCAs to be designated as supplementary rather than primary evidence [2,64], and why prospective hybrid trial designs—in which a small concurrent control arm supplements the synthetic arm—remain the most defensible near-term approach for regulatory submission [2,65].

## 7. Limitations, Ethical Considerations, and Algorithmic Bias

Despite the acceleration and efficiency that AI can provide to the pharmaceutical pipeline, the uncritical adoption of these complex technologies presents scientific, clinical, and ethical risks. AI models, particularly deep neural networks utilized in precision medicine, are fundamentally dependent upon the quality, breadth, and integrity of their underlying training data [66,67,68].

### 7.1. Data Bias and Demographic Underrepresentation

A primary limitation of AI in global healthcare is the potential perpetuation and algorithmic amplification of historical data biases [11,68]. Modern genomic databases and historical clinical trial repositories predominantly feature biological data derived from populations of European descent, while underrepresenting minority demographics, native populations, and female cohorts [68]. Consequently, AI models trained on these skewed datasets—whether utilized for target identification, de novo molecular design, or patient stratification—risk generating drug profiles that may fail to account for the pharmacogenomic variability of a diverse global population [68].

Furthermore, the subjective logic of developers in choosing proxy variables for training data can inadvertently result in algorithmic discrimination [58]. A landmark 2019 study highlighted a failure where an algorithmic model used widely to predict patients’ future health risks incorrectly equated historical medical costs with actual healthcare needs [58]. Because minority populations historically incurred lower healthcare costs due to systemic access barriers and socioeconomic disparities, the AI erroneously assessed them as healthier than white patients with identical disease severity [58]. Correcting this algorithmic bias was estimated to increase the proportion of minority patients receiving necessary additional care from 17.7% to 46.5% [58].

The magnitude of demographic underrepresentation in training datasets for AI-driven drug discovery is quantifiable and substantial. Genome-wide association study (GWAS) repositories remain disproportionately European-ancestry derived: as of 2023, approximately 78% of GWAS participants in the GWAS Catalog were of European descent, despite European populations constituting fewer than 16% of the global population. In multi-omics target identification models trained on these datasets, pharmacogenomic variant frequencies for CYP2D6 poor metabolizer alleles—which govern the metabolism of approximately 25% of clinically used drugs—are systematically underestimated for East Asian and African ancestry populations, potentially producing target prioritization models that deprioritize mechanisms of particular relevance to these groups. Structural remediation requires more than augmenting dataset size; it requires the architectural incorporation of ancestry-conditioned embeddings in graph neural networks used for target identification, the use of transfer learning from population-specific genomic cohorts such as the Africa Wits-INDEPTH Partnership for Genomic Studies (AWI-Gen) and the Saudi Biobank, and the adoption of fairness constraints during model training that penalize performance disparities across demographic subgroups. The Saudi Biobank, with over 200,000 participants and extensive phenotypic data, represents a particularly valuable and underutilized resource for recalibrating AI-driven discovery models toward Middle Eastern and Arabian Peninsula populations, for whom existing pharmacogenomic models have demonstrated significant predictive gaps.

Beyond racial and ethnic representation, sex- and gender-specific considerations represent a critical and underaddressed dimension of algorithmic bias in generative AI for drug discovery. Pharmacogenomic dimorphisms between biological sexes are well established and clinically significant: CYP3A4 and P-glycoprotein (P-gp) activity differences of up to 40% between males and females influence the metabolism and bioavailability of a broad range of therapeutics [68]. Furthermore, diseases prominently featured in AI-driven discovery efforts—including IPF and Alzheimer’s disease—exhibit well-documented sex-specific prevalence patterns, disease trajectories, and treatment responses [68]. Despite this, the majority of foundational genomic and transcriptomic databases used to train generative models reflect historical underrepresentation of female cohorts in clinical trials and biological repositories. Consequently, generative models trained on such data may implicitly encode male-normative pharmacological assumptions, producing molecular candidates and dosing predictions that are suboptimally validated for female patients. Addressing this gap requires deliberate architectural choices: incorporating sex as a conditioning variable within generative latent spaces (i.e., sex-conditioned VAEs or diffusion models), mandating sex-disaggregated performance benchmarking during model validation, and ensuring that training datasets are curated to achieve demographic parity across biological sex. Regulatory agencies and journal editors are increasingly demanding such sex-stratified reporting as a prerequisite for translational credibility, and the generative AI field must align accordingly.

### 7.2. Data Silos, Hallucinations, and the Need for Explainable AI

The modern pharmaceutical industry is inherently proprietary, resulting in fragmented, siloed data ecosystems [66,68]. The lack of standardized, interoperable data across international healthcare institutions restricts the input necessary to train robust foundational models [27]. When LLMs and generative architectures are forced to extrapolate from incomplete or siloed data, they become prone to “hallucinations”—the generation of confidently presented but factually incorrect biological data or clinical outputs [69,70]. In the context of automated pharmacovigilance or clinical trial summary drafting, an AI hallucination could obscure severe toxicity signals [69,70].

The term “hallucination” as applied to LLMs and generative models in pharmaceutical contexts encompasses at least three mechanistically distinct failure modes that warrant differentiated mitigation strategies. The first is factual confabulation—the generation of syntactically plausible but factually incorrect biomedical assertions, such as fabricated clinical trial outcomes, non-existent gene–disease associations, or erroneous drug–drug interaction claims. A 2025 systematic review of 128 studies on ChatGPT (versions 3.5 and 4) in health care reported substantial variability in accuracy across medical domains, with more inconsistent performance in clinical pharmacy and pharmacology than in other areas [69]. A separate systematic review and meta-analysis of drug-counseling applications of ChatGPT-4 found a pooled accuracy of 86%, implying a non-trivial error rate of about 14% in medication-related answers, with marked heterogeneity between studies [68]. Additional evaluations of clinical pharmacy tasks show that ChatGPT performs well in some counseling scenarios but is notably weaker for prescription review, adverse drug reaction recognition, and causality assessment, where accuracy scores can fall near 4–6 on a 10-point expert scale [71]. Collectively, these findings indicate that factual errors in pharmacotherapy-related outputs remain common and are more pronounced in complex or advanced decision-making contexts, underscoring the need for expert oversight before clinical use [69,70,71]. The second failure mode is structural hallucination in molecular generation—the output of molecules with valid SMILES notation but physically impossible or synthetically inaccessible structural features, such as pentavalent carbons or strained ring systems with no viable synthetic route. Estimated rates of structurally hallucinated molecules vary from 3% to 25% across generative architectures, with VAEs and GANs exhibiting higher rates than SELFIES-constrained models and diffusion approaches. The third mode is contextual misattribution, in which a generative model correctly identifies a chemical scaffold or biological target but attributes incorrect mechanistic properties based on spurious co-occurrence patterns in training data, a particular hazard when biomedical literature mining is used as a data source given the well-documented prevalence of inconsistent nomenclature and retracted findings in published pharmacological literature. Robust mitigation requires architecture-specific responses: retrieval-augmented generation (RAG) frameworks with curated pharmacological knowledge bases for factual confabulation; SELFIES-constrained decoding layers and synthesizability scoring functions for structural hallucination; and cross-referencing pipelines against curated ontologies such as ChEMBL (European Bioinformatics Institute (EMBL-EBI), Hinxton, UK) and UniProt (Swiss Institute of Bioinformatics (SIB), Geneva, Switzerland; European Bioinformatics Institute (EMBL-EBI), Hinxton, UK; Protein Information Resource (PIR), Georgetown University, Washington, DC, USA) for contextual misattribution.

To mitigate these risks, the integration of generative AI into clinical workflows requires rigorous human-in-the-loop oversight. However, clinical researchers warn of emerging phenomena termed “oversight fatigue” and “cognitive drift,” whereby human operators gradually become desensitized to AI outputs, trusting algorithms implicitly and failing to scrutinize erroneous data [57].

Addressing these challenges may require the adoption of XAI frameworks and strict adherence to FAIR (Findable, Accessible, Interoperable, and Reusable) data principles across both academia and industry [6,57]. Regulatory agencies, including the U.S. Food and Drug Administration (FDA), are actively defining validation protocols for AI-driven diagnostic and therapeutic models [57]. These frameworks mandate transparency in decision-making, ensuring that algorithms do not operate as opaque “black boxes” when consequential clinical decisions are at stake [57].

Importantly, the FDA’s 2024–2025 draft guidances on AI/ML-enabled medical devices and drug development tools have introduced more specific expectations for the validation and transparency of AI models used in clinical and regulatory decision-making contexts. These guidances emphasize the need for predetermined change control plans, performance monitoring frameworks, and the use of standardized benchmarking datasets to demonstrate model robustness and generalizability prior to regulatory submission. In the domain of generative molecular design specifically, initiatives such as the AI-Driven Drug Discovery Benchmark (AIDDB) and the Open Generative Molecular Design Challenge are emerging as community-endorsed evaluation frameworks, providing standardized test sets against which generative model outputs can be objectively compared across institutions. Adoption of such benchmarking suites would provide a clearer and more reproducible pathway for regulatory acceptance of AI-generated drug candidates, and their incorporation into development workflows is increasingly expected by both academic reviewers and regulatory bodies.

### 7.3. Generalization Failure and Distribution Shift

One of the most practically consequential but underappreciated limitations of generative AI in drug discovery is distributional generalization failure—the systematic degradation of model performance when applied to chemical or biological spaces that are underrepresented in training data. This is not a marginal concern: retrospective analyses of generative molecular design campaigns have found that between 20% and 40% of AI-generated hit molecules that achieve high predicted binding affinity scores subsequently fail in experimental validation due to distribution shift between the in silico training environment and the physical assay conditions [3,4]. Sources of this shift include differences between crystallographic protein structures used for training and the dynamic conformational ensembles present in solution; the systematic overrepresentation of ATP-competitive kinase inhibitor scaffolds in publicly available training datasets; and the near-complete absence of covalent binders, macrocycles, and PROTACs from most foundational training corpora, limiting the generative models’ capacity to explore non-classical modality spaces. Addressing generalization failure requires not only larger and more chemically diverse training sets, but also principled uncertainty quantification—the ability of a model to recognize and flag when a query molecule lies outside its reliable predictive domain. Bayesian deep learning and conformal prediction approaches offer promising frameworks for operationalizing predictive confidence intervals in molecular property models, but their routine integration into generative design workflows remains limited.

## 8. Conclusions

The integration of generative AI represents a notable shift in pharmaceutical research, contributing to the transition of the industry from empirical methodologies toward a predictive, data-driven innovation model. These technologies have demonstrated the capacity to identify novel targets such as TNIK in a fraction of the traditional timeline, generate optimized molecular entities de novo, and enhance clinical trials through medical LLMs and SCAs. However, the translational success of AI remains contingent upon overcoming systemic data biases, the risk of algorithmic hallucinations, and the need for greater model interpretability. The adoption of XAI and FAIR data principles, combined with rigorous human-in-the-loop oversight, will be essential to ensure that these computational tools provide safe, equitable, and transparent solutions for modern precision medicine. Future research should focus on prospective validation of AI-generated drug candidates in diverse patient populations, the development of standardized benchmarking frameworks for generative models, and the establishment of regulatory pathways that balance innovation with patient safety.

### Toward a Self-Improving Pharmaceutical Pipeline

The Generative AI Continuum framework articulated in this review implies a specific set of priorities for the field. First, prospective validation of AI-generated drug candidates in demographically diverse populations—including Middle Eastern, African, and South Asian cohorts currently underrepresented in pharmacogenomic databases—must become a prerequisite rather than an aspiration for translational credibility. Second, federated learning architectures offer a technically viable pathway for cross-institutional data integration that preserves data privacy while enabling the training of generalizable models across siloed repositories; their systematic adoption across academic medical centers and pharmaceutical partners warrants coordinated investment. Third, the co-design of regulatory and algorithmic validation standards—bringing together AI developers, regulatory scientists, and patient advocates in the drafting of benchmarking frameworks—is essential to ensure that the speed of methodological innovation does not outpace the institutional capacity to evaluate it responsibly. Together, these priorities define the translational agenda that must accompany the technical development of the Generative AI Continuum if its clinical promise is to be realized equitably and safely.

## Figures and Tables

**Table 1:** Summary of Deep Generative Architectures for De Novo Molecular Design.

Architecture Type	Mathematical Mechanism	Primary Representation	Strengths in Molecular Drug Design
Variational Autoencoders (VAEs)	Maps input data to a continuous probabilistic latent distribution via an encoder, from which new samples are decoded [4,5]	1D Strings (SMILES, SELFIES)	Continuous property optimization, multi-parameter conditioning, and smooth interpolation between known structures [4]
Generative Adversarial Networks (GANs)	Employs a zero-sum game between a generator creating molecules from noise and a discriminator distinguishing real from synthetic molecules [4]	1D Strings, 2D Graphs	Generates realistic structural distributions; useful for targeted library generation without explicit likelihood modeling [4]
Normalizing Flows	Learns exact, invertible transformations of simple probability distributions to model complex molecular datasets [3]	2D Graphs, 3D Point Clouds	Provides exact likelihood estimation, improving chemical validity of generated molecules [3]
Geometric Diffusion Models	Destroys data through a forward Markov chain of noise injection, then trains a network to reverse this process [4]	2D Graphs, 3D Point Clouds, Protein Sequences	State-of-the-art for generating precise 3D geometries and conditional ligand generation within protein pockets [3,8]

**Table 2:** AI Biomarker Discovery Frameworks for Precision Diagnostics.

Framework	Biological Modality	Algorithmic Mechanism and Clinical Output
MILTON	Proteomics combined with routine clinical markers	Augments traditional diagnostic data with AI-selected proteomics biomarkers to improve predictive performance across disease states [10]
PRSmix	Genomics (Polygenic Risk Scores)	Uses elastic net regression to aggregate and optimize polygenic risk scores, capturing epistatic interactions to improve genomic risk biomarkers [10]
EpiSign	Epigenomics (DNA Methylation)	Deploys SVMs to analyze methylation data, identifying episignatures associated with Mendelian disorders and generating methylation variant pathogenicity scores [10]
CytoTRACE2	Single-cell Transcriptomics	Uses interpretable deep learning based on Gene Set Binary Networks to predict responses to chemotherapy and immune checkpoint inhibitors [10]

## Data Availability

No new data were created or analyzed in this study. Data sharing is not applicable to this article.
